# Integrating Explainability into Graph Neural Network Models for the Prediction of X-ray Absorption Spectra

**DOI:** 10.1021/jacs.3c07513

**Published:** 2023-10-09

**Authors:** Amir Kotobi, Kanishka Singh, Daniel Höche, Sadia Bari, Robert H. Meißner, Annika Bande

**Affiliations:** † Helmholtz-Zentrum Hereon, Institute of Surface Science, Geesthacht, DE 21502, Germany; ‡ Helmholtz-Zentrum Berlin für Materialien und Energie GmbH, Berlin, DE 10409, Germany; § Deutsches Elektronen-Synchrotron DESY, Hamburg, DE 22607, Germany; ∥ Zernike Institute for Advanced Materials, University of Groningen, Groningen 9712, Netherlands; ⊥ Institute of Chemistry and Biochemistry, Freie Universität Berlin, Berlin, DE 14195, Germany; # Hamburg University of Technology, Institute of Polymers and Composites, Hamburg, DE 21073, Germany; ○ Leibniz University Hannover, Institute of Inorganic Chemistry, Hannover, DE 30167, Germany

## Abstract

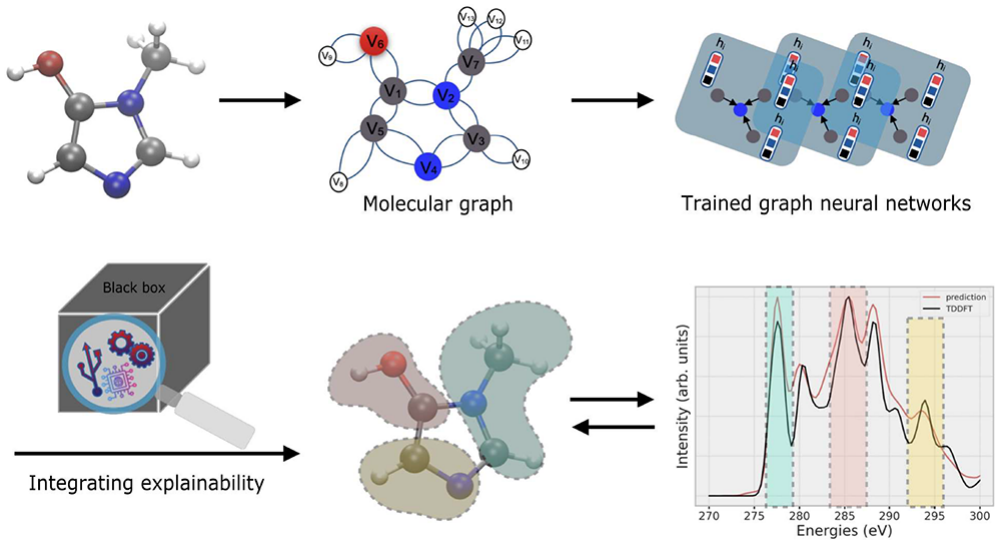

The use of sophisticated machine learning (ML) models, such as graph neural networks (GNNs), to predict complex molecular properties or all kinds of spectra has grown rapidly. However, ensuring the interpretability of these models’ predictions remains a challenge. For example, a rigorous understanding of the predicted X-ray absorption spectrum (XAS) generated by such ML models requires an in-depth investigation of the respective black-box ML model used. Here, this is done for different GNNs based on a comprehensive, custom-generated XAS data set for small organic molecules. We show that a thorough analysis of the different ML models with respect to the local and global environments considered in each ML model is essential for the selection of an appropriate ML model that allows a robust XAS prediction. Moreover, we employ feature attribution to determine the respective contributions of various atoms in the molecules to the peaks observed in the XAS spectrum. By comparing this peak assignment to the core and virtual orbitals from the quantum chemical calculations underlying our data set, we demonstrate that it is possible to relate the atomic contributions via these orbitals to the XAS spectrum.

## Introduction

X-ray absorption spectroscopy (XAS) is an important characterization technique in chemical analysis to unveil the atomic structure of matter, having a broad range of applications in material science,^1^ biomedical research,^2^ and identification of metals and solids.^3^ XAS is particularly useful in the investigation of the electronic and geometric structure of biomolecules, nanoparticles, and metal complexes.^4−6^ The interpretation of experimentally obtained XAS spectra is, however, complicated due to the intricate interplay between the complex electronic structure of the material and the adsorption of X-ray photons. Several factors, including the chemical environment of the atom, the presence of solvents, and the energy of the incident X-rays, influence this complexity.^7^ Therefore, sophisticated—but computationally also expensive—theoretical methods from *ab initio* quantum chemistry can accurately predict XAS and are a necessary complement to interpret experimental results.^8^

Machine learning (ML) techniques are being increasingly applied to various areas of theoretical and computational chemistry given their ability to infer structure–property relationships on the basis of large amounts of data.^9−11^ Among those ML techniques, graph neural networks (GNN) and deep neural networks (DNN) are promising candidates to predict the properties of matter, such as the electronic structure,^12^ at a higher computational speed, already making them favorable for high-throughput calculations in materials design and drug discovery.^13,14^ Thus, the ability to perform efficient computations with high accuracy has demonstrated that ML techniques are advantageous in domains such as various types of spectroscopy, including vibrational and optical.^12,15−24^

Several studies have focused on X-ray spectroscopy using ML methods with the additional aim to improve the understanding of the contribution of different atomic environments to the peaks occurring in the spectra.^22−24^ Accurate prediction of XAS spectra has been accomplished by employing some of the more sophisticated ML models, such as GNNs and DNNs.^17,25,26^ However, a large number of layers in the underlying neural network, as well as a high parameter count, implies such models are black-box,^27^ which means understanding the rationale behind predictions is a challenging task. On the other hand, ML models designed to predict XAS spectra must provide clear peak assignments, as this option for interpretation is typically required in spectroscopy experiments and often necessitates theoretical calculations. The comprehensibility of why ML models can achieve this peak assignment capability must be transparent to users to ensure trust in the predictions, given the diverse range of applications of XAS in material and biochemical sciences.^7,28,29^ It is therefore imperative to develop an understanding of the XAS predictions made by complex ML models and ascertain whether the predictions align with human logic and decision-making, as incorporated in the quantum-mechanical equations. This can be achieved using explainable artificial intelligence (XAI) methods, which provide a window into the ML model’s decision-making process and correlations uncovered by the model through data analysis.^30^ Justification and interpretability offered by XAI methods not only provide evidence defending why a prediction is trustworthy with quantitative metrics but also refer to the degree of human understanding intrinsic within the model.^10,31,32^

Numerous techniques are available to incorporate explainability in GNN and DNN models.^33,34^ Our emphasis in this work lies in using a method known as attribution.^35^ Attribution methods have found widespread use in applications where the input data consists of images or text, composed of features such as pixels, characters, and words.^36,37^ In these cases, attribution scores highlight particular regions via pixels of the image or certain characters or words in a text that affect, in this case, the decision-making of the ML model used in the task. Therefore, it is relatively easy to validate such explanations in image- or text-based tasks. However, validating explanations for chemical property prediction is challenging since a property is often the result of a complex interplay between the geometric and electronic structure of the atoms in a molecule. This gives rise to intricate structure–property connections within molecules, especially complex properties such as X-ray absorption spectra, which only find interpretation by the examination of each individual peak detected through a combination of experiments and simulations.^21^ Therefore, the validation of explanations generated using attributions also requires the creation of a robust “ground-truth” benchmark using such domain-specific knowledge, which is often a challenging task in molecular-property prediction. Examining the robustness of GNN models to predictions on unseen data, being possibly biased toward specific chemical structures, is yet another challenge in understanding the overall performance of different models.^38,39^

In this study, we introduce a framework that uses a combination of graph attributions and ground-truth data generated from linear-response time-dependent density functional theory (TDDFT),^40^ to provide explainability on GNN models trained to predict carbon K-edge XAS spectra of organic molecules. Carbon K-edge spectroscopy was used for XAS for various reasons. First, carbon atoms play a central role in the structure and function of a wide range of organic molecules as well as inorganic materials. Second, carbon K-edge XAS offers a unique perspective, providing valuable insights into the structure, function, and reactivity of these molecules.^41,42^ Finally, among the XAS calculations, K-edge spectroscopy on a main group element is less complicated than, for example, the spectroscopy on the transition metal L edge, and can be computed via TDDFT on a time scale that allows the creation of a large data set. To train the different GNN architectures, an in-house QM9-XAS data set, based on a subset of the QM9 data set or organic molecules,^43^ was set up (see Data Availability Statement). We compare the performance of the trained models in predicting XAS spectra on the test data set. In order to evaluate the explainability of GNN models, we analyze the ability of these models to identify the contribution of atoms and their surrounding environment toward the distinct peaks in the XAS spectrum. For creating the “chemical” ground truth pertaining to XAS, we created a data pipeline that inputs the output of TDDFT calculations and renders the labels to atoms, indicating whether or not an atom contributes to a specific excited state in XAS. These ground-truth values are then finally quantitatively compared with the attribution scores obtained from GNNs. Applying this method to different GNN models, we find that specific GNN architectures, which incorporate both global and local information on atoms, offer superior explanations for the peaks observed in the carbon K-edge XAS spectra. Additionally, we investigate the robustness of the GNN models by randomly perturbing molecules in the test data set, to rationalize the difference in the explainability power of various used GNN architectures.

## Methods

### The QM9-XAS Data Set

While X-ray absorption spectroscopy is a popular technique in chemistry, to the best of our knowledge there is no organic molecule XAS data set that is large enough and available for training ML models. Therefore, we used the QM9 data set^43^ containing 132,531 organic molecules composed of the first and second row of main group elements H, C, N, O, and F. We choose a random subset of the QM9 data set, containing 56,000 molecules, which we term QM9-XAS for the purpose of our data set. We use these structures to calculate carbon K-edge XAS spectra with the time-dependent density functional theory (TDDFT)^44^ method, which is in general a useful complement to experiments and allows for the interpretation of spectral peaks. More specifically we used the ORCA electronic structure package^45^ to calculate TDDFT at the B3LYP/TZVP^46,47^ level of theory. All calculated XAS spectra were obtained in the energy ranges *E*_min_ = 270 eV and *E*_max_ = 300 eV and peaks broadened using Gaussians of widths 0.8 eV. The resulting curves were discretized into *N*_grid_ = 100 points between. This step ensures that the length of the target output to be learned for ML applications is consistent across all spectra. Further processing is then performed to generate tuples of molecular graphs and their spectra to convert them into a format optimal for training GNN models. Molecular graphs were generated from the SMILES strings of the molecules, which were available in the original QM9 data set using the RDKit^48^ python library. Since our models are implemented using the Pytorch Geometric^49^ library, the graph and spectrum tuples were converted into the native data set class of this library.

### Graph Neural Networks

GNNs are neural networks specifically designed to treat unstructured molecular data.^50^ A graph is formally defined as a tuple of *G* = (*V*, *E*) of a set of nodes *v* ∈ *V* and a set of edges *e*_*v*,*w*_ = (*v*, *w*) ∈ *E*, which defines the connection between nodes. It is intuitive to represent molecules as graphs, in which atoms and the bonds between them are represented as nodes and edges, respectively. Further information about each atom and bond in a molecular graph is incorporated in the form of node and edge feature vectors added to the tuple *G* of each graph in the data set. A node (atomic) feature vector represents information such as the atom type (e.g., C, H, N, O, or F) or the number of hydrogen atoms attached to it. Similarly, edge (bond) feature vectors are representatives of properties such as the bond lengths between two atoms or the bond multiplicity. We employ one-hot encoding to convert most of the node and edge features, including categorical attributes, such as atom type, into numeric vectors. All encodings used in this work are summarized in Table 1.

**Table 1:** Features of Nodes and Edges (Atoms and Bonds) as Represented in the Encoded Vector in Conjunction with Their Respective Type of Encoding

Node feature	Encoding
Atomic number	One hot
Hybridization	One hot
Aromaticity	One hot
Number of H atoms	Integer

Edge feature	Encoding
Bond distance	Real
Bond type	One hot

A GNN layer takes as input a graph with node and edge features and outputs a graph with the same topology where the node, edge, and global graph information is updated. To achieve this, the node and edge information represented as feature vectors are first converted into vectors in higher dimensional space (feature space) referred to as node and edge states, respectively, using a transformation function. Transformation functions can be fully connected layers, convolutional layers, or recurrent layers depending on the GNN architecture. A fundamental part of GNNs is the so-called propagation (or message-passing) process used to update these node (or edge) states. Message passing occurs in two steps: The first step involves gathering the information on the nodes (or edges) surrounding a target node by collecting their node states. In the second step, these states, along with the state of the target node, are aggregated using an aggregation function such as sum or average. If the final task is to predict the property of a graph, then these updated node states are further aggregated using a graph-level aggregation function, termed readout.

Different GNN architectures have different message propagation and readout functions that affect the node, edge, and graph states obtained at the end of a message-passing process. In this work, we trained ML models on three GNN algorithms. The first architecture is the graph convolutional neural network (GCN),^51^ which employs only node states to aggregate information in the message-passing process. The second GNN model is GraphNet,^52^ in which a global state vector, including node and edge states, is used in the message function. The third is the multihead graph attention network (GATv2)^53^ in which an attention mechanism^54^ is used to aggregate node information. The attention mechanism in GATv2 allows for the calculation of edge weights to each node in the neighborhood of a target node, which assigns an importance value to the message passed from each node to the target node. Training a multihead GATv2 converges faster at a moderately higher computational cost, while also increasing the robustness of the final model since it is in principle trained on multiple attention instances in parallel.

### Training

In order to assess various trained models, the QM9-XAS data set was shuffled and divided into a training set of 50k samples and a test set of 6k samples, with the training data further partitioned into an 80:20 ratio for training and validation. The GNNs and all fully connected layers were trained for 1000 epochs, at a learning rate of 1 × 10^–3^, and a batch size of 100 samples. A learning rate scheduler was implemented to reduce the learning rate by a factor of 0.8 every 100 epochs. For all the models, three GNN hidden layers with sizes of 128, 256, and 512 were used for node updates, and a fully connected layer was used as the output layer for predictions. We used the AdamW optimizer^55^ and the root mean squared error (RMSE) as the loss function to train the models. In order to keep track of overfitting, we monitor the RMSE loss on the validation set after every 50 epochs. All models were trained on a single NVIDIA Tesla A100 64GB GPU. We select the model which has the best RMSE loss and relative spectral error (RSE)^19^ on the validation data set.

RSE is obtained by dividing the RMSE among the target *y*^tar^ and the predicted *y*^pred^ intensities of the signal at energy *E*, by the total spectral energy of the target. In the discretized spectrum in steps of Δ*E* = (*E*_max_ – *E*_min_)/*N*_grid_, the RSE is approximated as
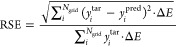
1A small relative spectral error indicates that the predicted spectrum is a good prediction of the original spectrum. The quality of XAS spectra predictions made by different GNN architectures was compared by calculating the average RSE on the test data set.

### Graph Attribution

Attributions or feature attributions are one of the most popular techniques used to explain the model’s predictions.^56^ The attribution method assigns scores to each input feature that reflects the contribution of that feature to an ML model’s prediction, thereby explaining the role played by that feature in the prediction.^38,57,58^ In the case of GNNs, attribution methods assign attribution scores to graph nodes and edges based on their contributions to the final prediction of the model. One way to visualize the attribution scores obtained is by overlaying a heat map on top of a graph, highlighting the importance of individual atoms to the target property in the case of a molecular graph. From these heatmaps, one can deduce structural correlations between the model’s rationale for good or bad predictions and compare them to existing knowledge of why the prediction should be so. GradInput (GI),^59^ class activation map (CAM),^60^ and gradient class activation map (GradCAM)^61^ have been shown to successfully explain predictions made by GNNs for molecular structure–property prediction models;^35^ that is, they can reveal the contribution of individual atoms or atom pairs to the model’s decision. Although GNNs and their interpretation through attribution techniques have proven successful in decoding binding mechanisms and performing materials discovery,^38,58,62^ to the best of our knowledge, these explainability techniques have not been employed in XAS analysis. The scoring attribution of atoms arising from CAM is intuitively well-suited to the phenomenon of XAS, where peaks in the spectrum arise from the local and global environments of atoms in a molecule.^63,64^

We, therefore, use CAM to obtain atomic contributions to the XAS spectra of molecules in the QM9-XAS data set to explain the spectrum predictions of GNN models. CAM attributions calculate the node weights *v*_*i*_ for highlighting the contribution of various nodes of the graph to the prediction. As discussed above, GNNs that perform property prediction on graphs use a global aggregation layer or a readout layer prior to the output layer. In our case, the model generates 100 values for the final spectrum by utilizing a layer consisting of 100 units (neurons). For the purpose of evaluating the attributions, each of these values can be treated as an independent class. CAM operates on the aggregation layer prior to this final layer and obtains attributions for these different “classes”, giving an insight into atomic contributions at each point in the spectrum. To compute CAM weights of a node for each class, let *F*_*k*(*i*)_ be the activation of a unit *k* in the last GNN convolutional layer, preceding the output layer, at node *i*. The CAM score at a node for a class *c* then is defined as^65,66^
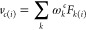
2where ω_*k*_^*c*^ denotes the weight of unit *k* for class *c*. Using this formulation, one can obtain CAM scores for each point in the spectrum of a given input molecular graph.

### Ground-Truth Evaluation

**Figure 1:**
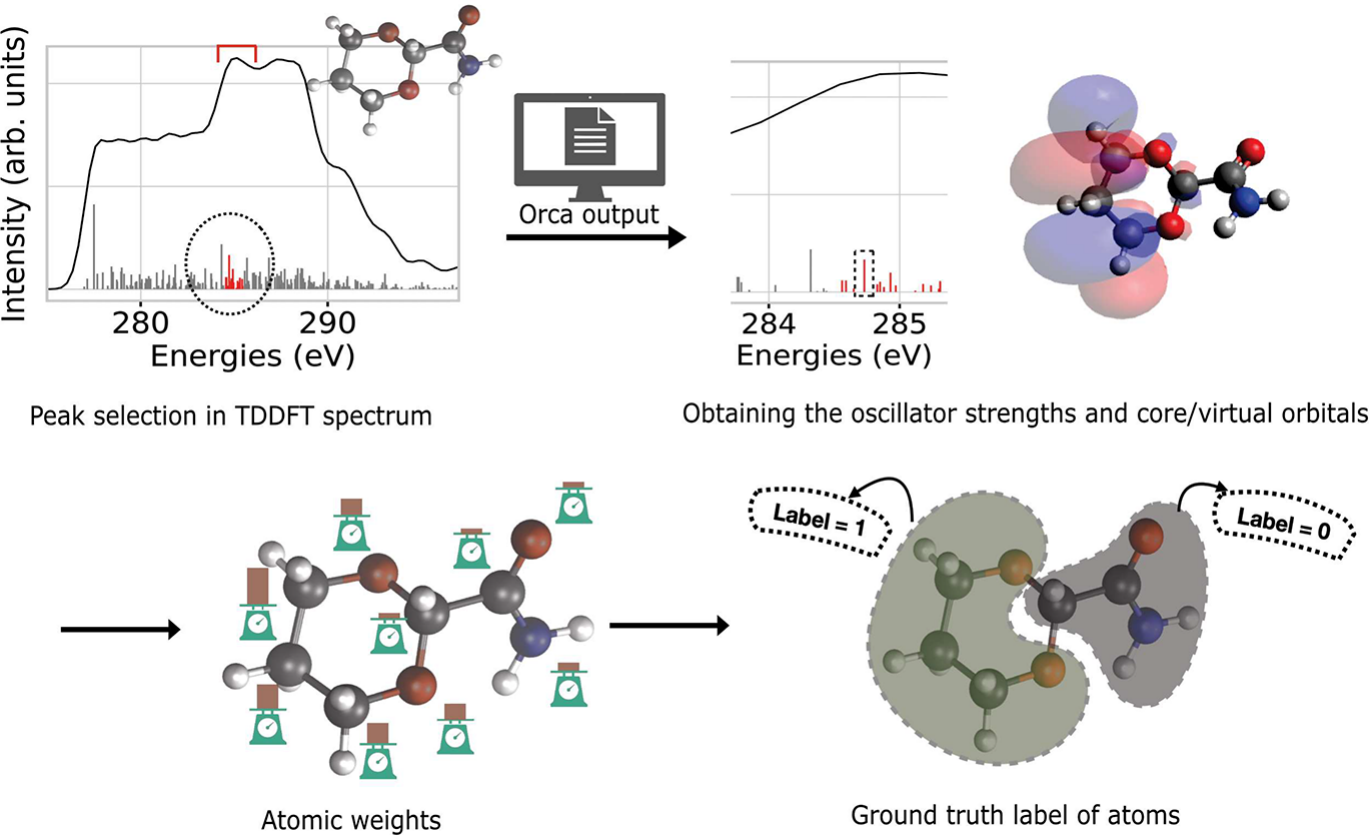
Ground-truth evaluation is based on TDDFT data. The process of evaluating the TDDFT data starts with selecting a specific peak in the XAS spectrum. The oscillator strength and orbital contributions for each excitation state in the peak are used to determine the final atomic contributions to the peak. Atoms in the molecule are then labeled based on the calculated weights, i.e., 1 for atoms contributing to the peak and 0 otherwise.

In addition to the evaluation of attributions, it is crucial to establish a ground-truth logic that enables the assessment of attribution quality. Hence, the agreement between CAM weights of the model’s prediction and ground-truth logic should be quantified. To this end, a definition for a numerically measurable ground truth for the excitations underlying the spectra is needed. In other instances of XAI in chemistry, a suitable ground truth was developed by directly considering the molecular fragments or functional moieties that experts knew to be important for decision making,^67^ such as binding mechanism learned by DNNs.^38^ Nevertheless, when it comes to predicting XAS, comparing attribution scores to ground truth becomes more complex, since it necessitates careful examination of all atoms in the molecule and a comprehensive understanding of the quantum mechanics behind X-ray excitations. Furthermore, delocalized molecular orbitals present yet another challenge for understanding the precise contribution of atoms to virtual orbitals in excitation states of XAS.^68^ Therefore, we have developed a method that assigns the ground-truth contributions of various atoms in a molecule to a peak in the TDDFT spectrum. It uses a combination of orbital populations of all of the initial and final states underlying the respective X-ray excitations and their oscillator strengths to obtain the contribution of each atom to a specific peak in the XAS spectrum. To derive atomic contributions in the ground truth, we first compute the core excitations within this energy range and then determine the atoms contributing to both the core and virtual orbitals of a certain excitation state. The atom contributions were weighted according to the oscillator strength of the corresponding excited state as well as the atom population per molecular orbital. In cases where the calculated weights in the ground truth necessitate the presence of particular atoms in a peak of the XAS spectrum, we label those atoms as 1 and all other atoms as 0. Figure 1 depicts the process of obtaining ground-truth labels for atoms. Given the fact that the optical transitions obtained from TDDFT are discrete lines and that the ML spectra are distributed on a grid and have wide peaks, for the comparison it is necessary to unify all CAM scores of a given peak to a given line from TDDFT spectrum. Hence, we summed up the CAM scores of all atoms in the molecule for all energy points in a range equivalent to the full width at half-maximum of a peak.

### Model Explainability

Explaining a model’s predictions involves comparing the ground truth to the attributions obtained from the model by using an XAI method. To measure to what extent our ML models learn the correct atomic contributions to the XAS spectra, we use the area under the curve (AUC) of the receiver operating characteristic (ROC).^38,69^ The ROC itself is a curve formed by plotting the rate of true positive outcomes and that of false positive ones at various classification thresholds that divide the assignments between the true and false classes. A true positive outcome occurs when a model tasked with distinguishing two or more classes correctly predicts the class to which an instance belongs. In our case, the CAM weight assigned to an atom at a certain peak matches the ground truth of the atoms belonging to an orbital. Similarly, a false positive occurs when the class under investigation is incorrectly predicted by the model, i.e., when atom contribution in ground truth and CAM disagree. The AUC thus quantifies the performance of a classification model into a single value between 0 and 1, where an area of 0.5 means that a model works only as good as a random classifier. A value of 1.0 means that the model has the ability to perfectly discriminate among different classes. In this particular case, the AUC is indicative of whether the model can correctly identify whether an atom contributes to a peak in the spectrum or not.

**Figure 2:**
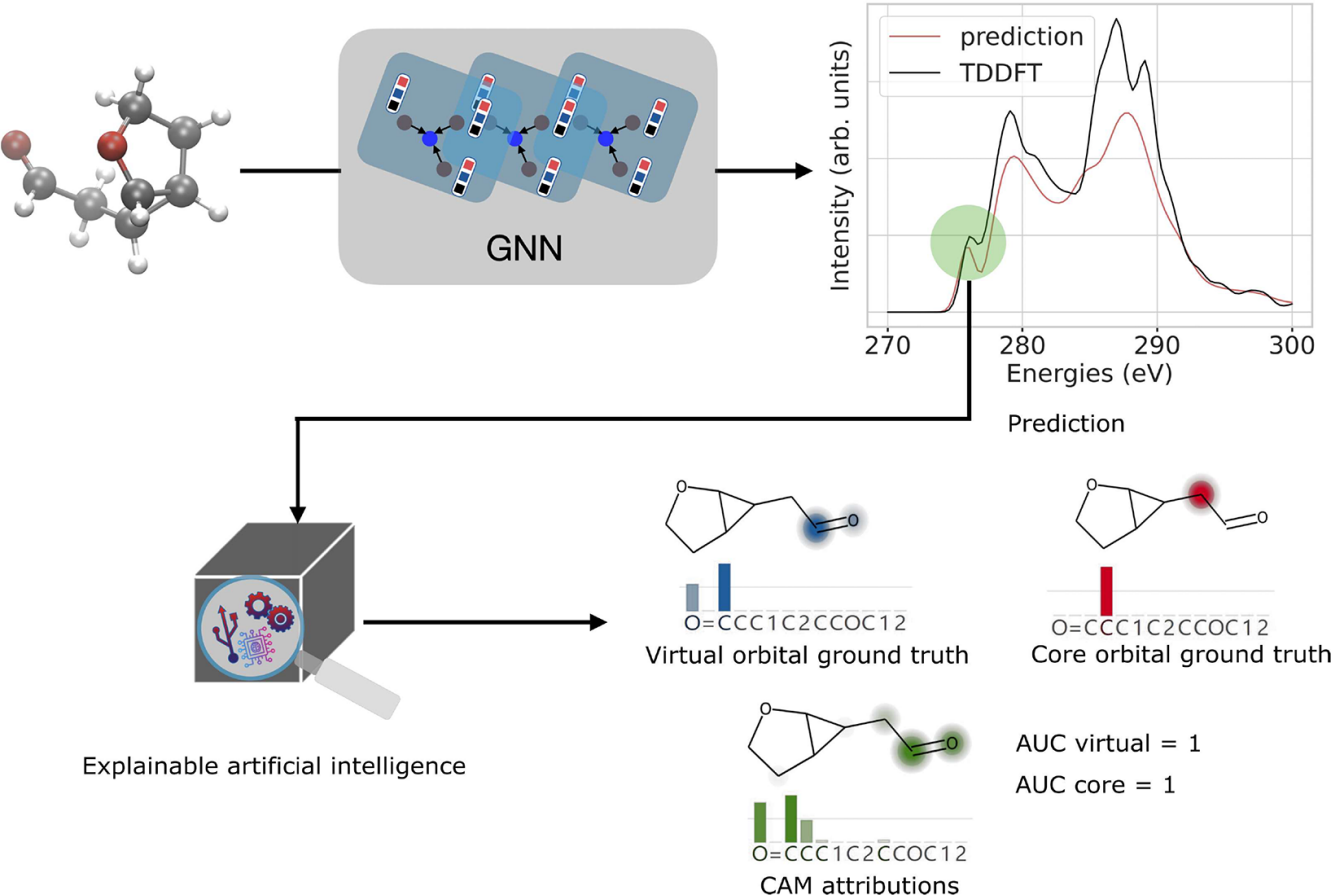
Workflow of the ML and explainability of the XAS spectrum. This process consists of converting a molecule to a molecular graph, training a GNN, comparison of the ML predicted and TDDFT spectra for obtaining the RSE, and finally applying the XAI technique to obtain here the CAM weights (green). In this example, the CAM weights are compared to ground-truth attributions for core (red) and virtual (blue) orbitals at the highlighted 277 eV peak of the spectrum, using a heatmap^70^ on the molecular structure. These ground-truth labels are then compared to CAM weights, giving the AUC values for the core and virtual contributions.

Figure 2 illustrates the workflow to make a GNN prediction of a spectrum, determine the CAM attribution, and compare it in the last step to the ground truth, i.e. the contribution of atoms to core and virtual orbitals obtained from TDDFT, here shown for a prediction made by the multihead GATv2 model. More explicitly, a model with a large AUC close to 1.0 would perfectly assign labels 0 and 1 to each atom in the spectrum for all of the molecules in the test set. Moreover, we identify the baseline of AUC as 0.5, which is basically a model classifier that randomly assigns these labels to the atoms in a molecule.

We compute attribution AUC values at each peak in a TDDFT spectrum and average them over all of the peaks to arrive at a final score that explains the degree of agreement between ground-truth logic and CAM attribution scores. The AUC is determined for different model architectures. To demonstrate that the explainability method is stable, we perturb a randomly chosen set of molecules from the test data set and evaluate the change in attribution AUC.

## Results and Discussion

### Model Performance

**Figure 3:**
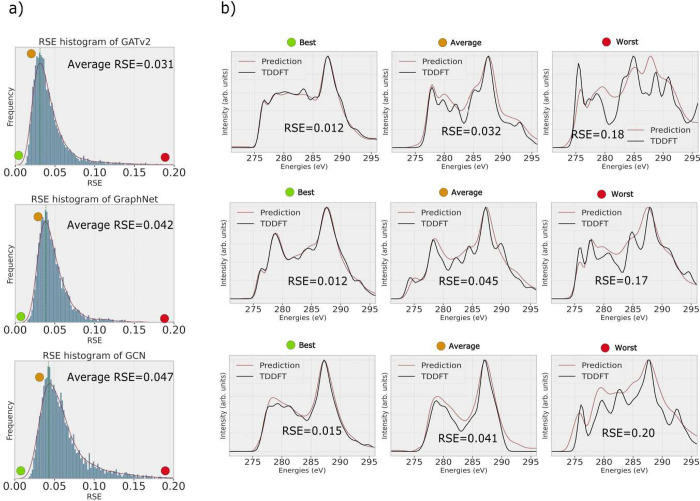
Evaluating the performance of various GNNs on the test data set. RSE histogram for all GNN models (a). While average RSE performances are close, GATv2 has a more left-skewed histogram distribution, indicating better performance over large portions of the data. Best, worst, and average predictions of the three GNN models with their respective RSE values (b).

To first visualize the predictions made by these GNN models, the best, average, and worst predictions of the XAS spectrum are demonstrated for each model based on RSE values in Figure 3b. While the best prediction across all models is a near-perfect replica of the TDDFT spectrum, the average and worse ones predict general features of the spectrum correctly, but miss out on the finer peak structure or incorrectly predict peak intensities. In Figure 3a, all RSE values for one model are plotted in a histogram and the average RSE is determined. The GATv2 model has a slightly lower average RSE value of 0.031 compared to 0.042 for GraphNet and 0.047 for GCN. The distributions look similar. They have their onset with a small slope at RSE = 0.0 and then quickly grow to their maximum around the average RSE. The decline is slow following the shape of a skewed distribution with a long tail leading to a low number of structures with RSE values above 0.1. Such structures are fewer for models with the GATv2 and the GraphNet GNN architectures, demonstrating their superiority for XAS predictions compared to GCN.

The above results are consistent with the findings of earlier research, which suggest that integrating an attention mechanism^54^ and applying combinatorial generalization,^52^ i.e. enabling the network to reason about the global structure of a graph, while learning the graph representation, as done in the GATv2 and GraphNet models, help enhance the learning of target properties related to both local and global structures of the graph.^71,72^ In the case of the GATv2 model, computing the importance of the neighboring atoms for a target atom in a molecule using the weighted attention mechanism assigns relevance to a local region of the molecule to a specific excitation energy in the spectrum, which differs from that of traditional GCN layers with fixed weights for connections between atoms. On the other hand, by incorporating relationships and interactions among nodes, edges, and global graph attributes, GraphNet significantly improves the acquisition of structure-properties relationships in XAS spectra.^52^

### Explainability of XAS Predictions

**Figure 4:**
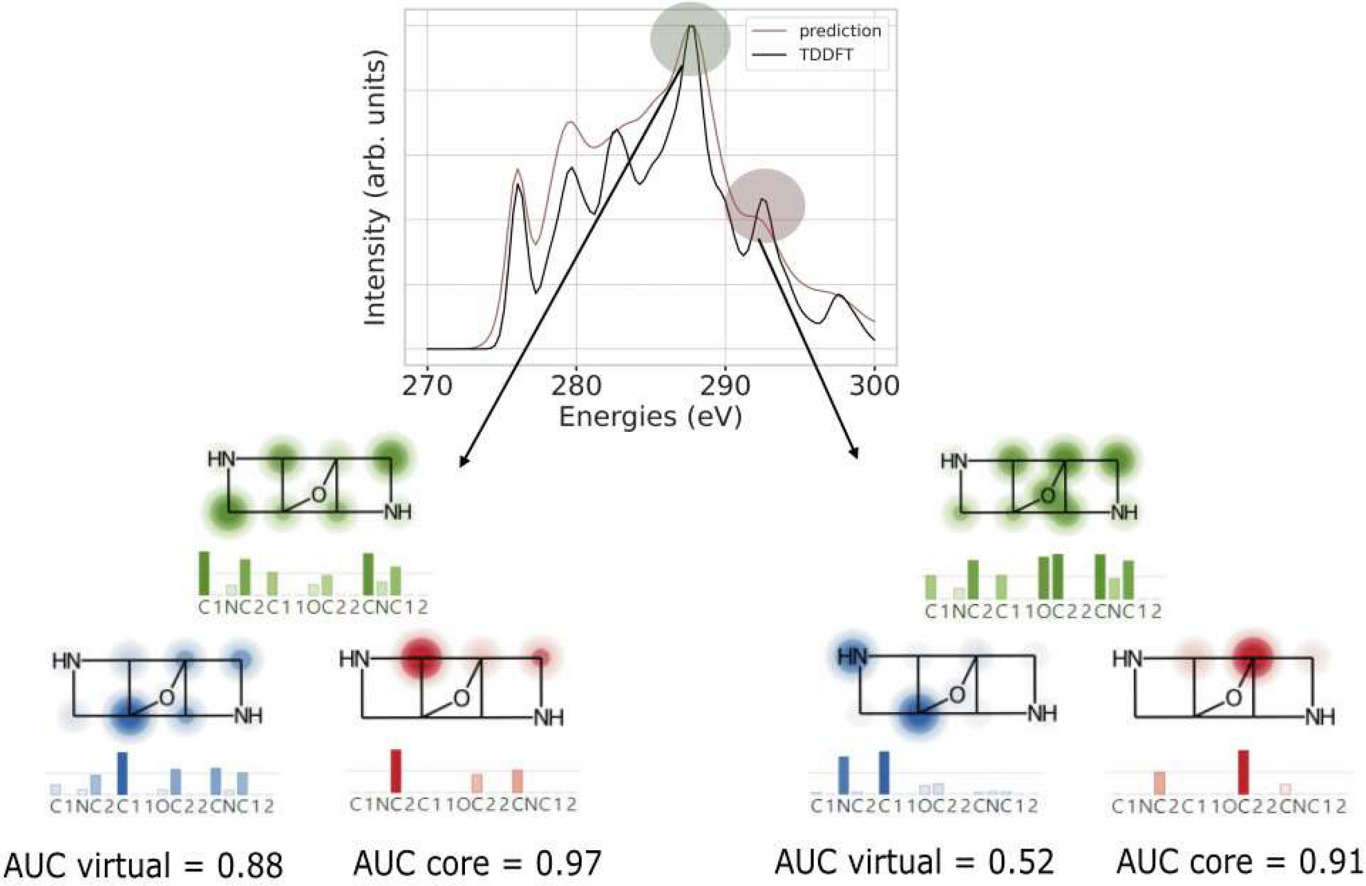
Attributions (green) are compared with the ground truth of core (red) and virtual (blue) orbitals via AUC values for two peaks of an XAS spectrum predicted by the GATv2 model. The model has higher AUC values when a peak in the predicted spectrum follows the TDDFT result.

**Figure 5:**
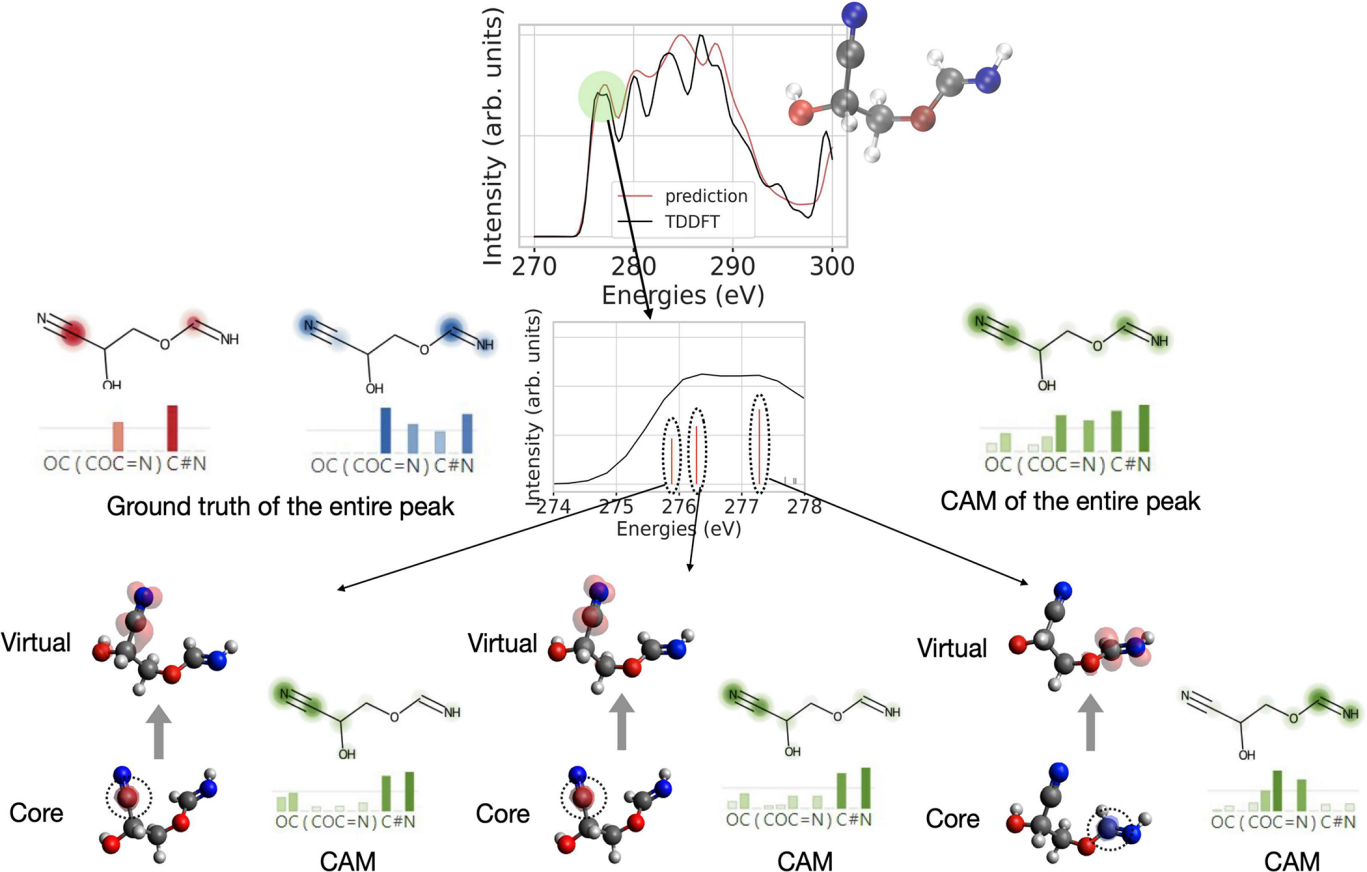
Exploring the correlation between CAM attributions of atoms and transition densities of a peak in the XAS spectrum. CAM attributions (green) and transition densities of three excitation states are visualized for a sample molecule in the test data set in the bottom part of the figure. The transition densities highlight the starting C core orbital, which is encircled for better visibility, in the bottom, and above the virtual orbital on the cyanide group for the two lower-energy peaks and the amide group for the third peak. The overlay of the three transition densities for the core (red) and the virtual (blue) states are shown on the left side of the close-up spectrum, while on the right side, the CAM of the entire peak is shown.

While comparing the prediction performance of different ML models is crucial, the similarity observed in the RSE distributions in the previous section motivates exploration of the interpretability of these models. Figure 4 illustrates the peak assignment via core and virtual orbitals from the TD-DFT calculation as red and blue spheres on participating atoms and via the CAM scores given as green spheres. The AUC values for the respective orbitals quantify this assignment. We compare an accurate GATv2 prediction at about 288 eV, in which the intensities of both curves lie on top of each other, with one with a larger deviation from the TD-DFT data at about 292 eV. In both cases, the core orbitals are accurately matched by the CAM score giving AUC values of above 0.9, significant quality differences occur for the virtual orbitals. Those contribute the most to an XAS spectrum in general. Hence, a good prediction comes with a good assignment of the peak with a large AUC of 0.88 eV. By contrast, the poorer XAS prediction with about 10% peak intensity differences also leads to a much reduced AUC of 0.52 only. In this case, one can already visually see that the CAM is much more significantly spread over the entire molecule, while the orbitals contributing are based only on two atoms, of which one is not a part of the CAM at all. Figure 5 gives a close-up visualization of the derivation of the CAM and the core and virtual orbital ground truth, by relating both to local excitations and the latter also to orbitals relevant to the respective excitation. This is done for the first three excitation states of the TDDFT calculation underlying the first signal of the broadened spectrum. Note that later signals are composed of a much larger number of transitions, making the visual comparison very cumbersome. We observe that the first two peaks originate from a transition of an electron on the cyano carbon atom to one of the π* orbitals of the CN group. This is exactly reflected in the CAM weights obtained at exactly the transition energy. The CAM weights show a low contribution at other atoms, which is insignificant. The third peak belongs to the *s* → π* transition on the amide group at the other end of the molecules, which is likewise highlighted by the local CAM. The total CAM overlays both transitions, and likewise does the ground truth of the contributing core (red) and virtual (blue) orbitals highlight the two C atoms or multiple bonds, respectively.

**Figure 6:**
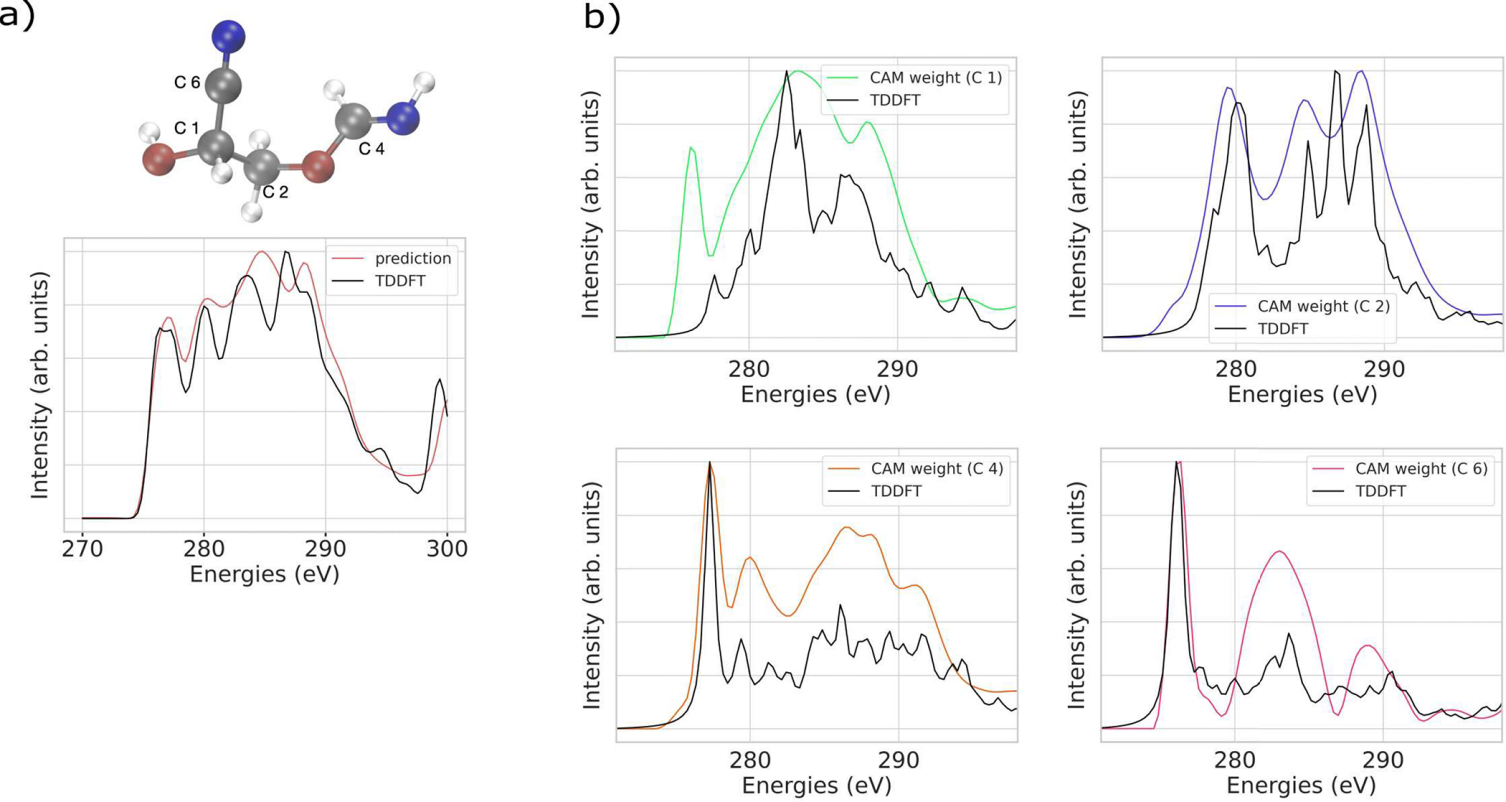
TDDFT (black) and GATv2 (red) predicted C K edge XAS spectra for an entire sample molecule (a). Calculated local XAS spectra (black) and CAM attribution weights (multiple colors) of individual carbon atoms in the molecule (b).

To further analyze the explainability of our best GNN model (i.e., GATv2), we performed TDDFT calculation of local atom XAS spectra of individual carbon atoms of a sample molecule in the test data set with the CAM attribution weights assigned to these carbon atoms for which the comparison is displayed in Figure 6. The CAM attribution weights, which are energy-dependent and hence appear as spectra in themselves, exhibit a reasonably accurate alignment with the main features of localized XAS spectra, although they do not entirely replicate all the peaks. In particular, CAM attribution weights of the carbon C1 next to the hydroxy group appear to show discrepancies, which can be due to the attribution technique or weaknesses in the model’s explainability concerning this specific atom. Although training a GNN model using localized XAS spectra to predict the spectra of individual carbon atoms is achievable and could potentially enhance the alignment between TDDFT and ML in terms of spectral shape and CAM attribution, generating a data set with atom-localized spectra through various methods requires more computational resources. CAM attributions of atoms from a complete molecular spectrum can provide an opportunity for creating a data set of localized spectra based on arbitrary XAS methods. Moreover, since the ultimate goal is to compare the predicted XAS to experimental spectra, training a model based on entire XAS spectra in certain energy ranges is more favorable.

**Figure 7:**
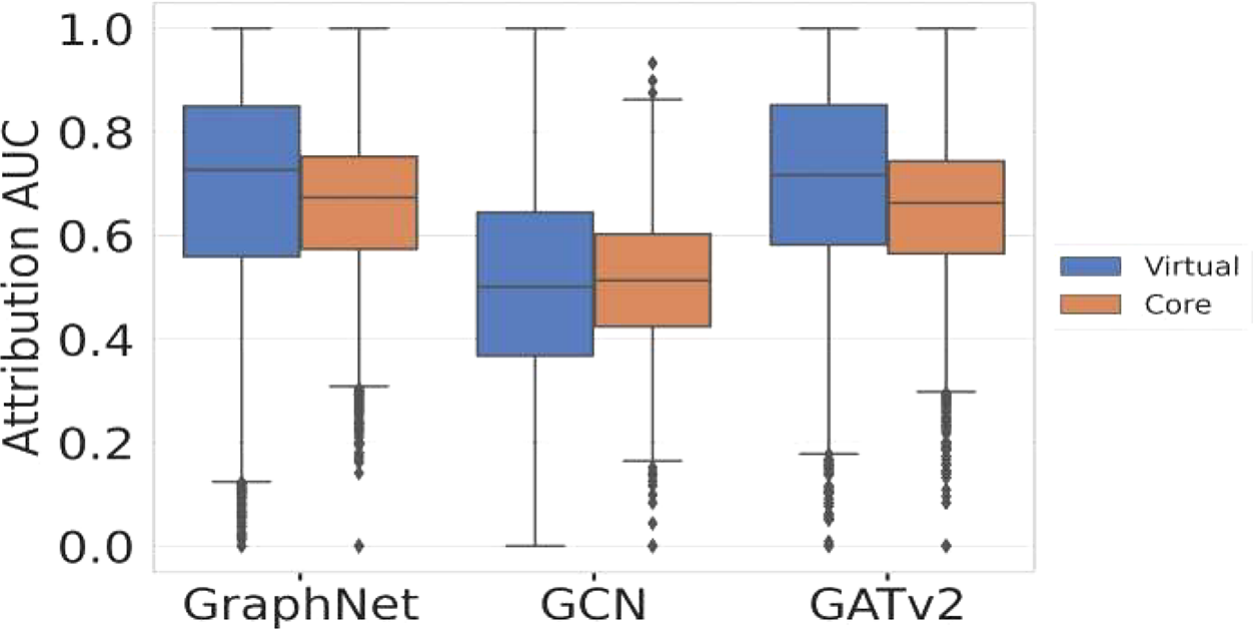
Attribution AUC score boxplots for the core and virtual orbitals of the three GNN models. The vertical line within the box indicates the median AUC value on the test data, while the length of the horizontal lines indicates the variance in AUC values for each model. Points beyond this range are considered outliers.

With this rationalization, the next step is to evaluate the attribution quality overall over the entire data set. Figure 7 shows box plots of the attribution AUC for core and virtual orbitals of the three GNNs evaluated over the full test data set. As seen from the figure, the GCN model gives an average attribution AUC close to 0.5, which means that the model barely outperforms a random classifier. This combination of good spectra predictions on the test data, as shown in Figure 3, and low average attribution AUC value by the GCN model is in line with a previous study, suggesting that the combination of near-perfect model performance and low attribution AUC indicates that the model fails to learn the ground-truth logic.^38^ In contrast to this, the GNN models with multihead GATv2 and GraphNet layers have a superior agreement with our developed ground-truth logic, with median values greater than 0.7 for both virtual and core orbitals. As a general trend, we also observe that the spread of core AUC values is lower across all models, while the AUC values for virtual orbitals are more widely spread out, as indicated by the high variances in the figure. Nevertheless, it should be noted that within the presented approach we are not able to learn to distinguish between the more localized core orbitals and the more delocalized virtual orbitals, which could be useful information for the model to be included. Models that have higher attribution AUC values for core and virtual orbitals, i.e., GraphNet and GATv2, demonstrate a greater ability to comprehend the contribution of atoms to the excitation energies of the XAS spectrum. GraphNet models associate and encode global graph context in addition to the message-passing on node and edge level, and this perhaps positively influences CAM attributions, giving them information beyond the local environment. Given that the peaks in XAS analysis are highly dependent on the local geometric and electronic structures of atoms,^73,74^ incorporating the interdependence of nodes and the global information on the molecular graph in GNNs, as done in these models, can facilitate capturing complex relationships between atomic coordination and specific excitation states in the XAS spectrum within its ML prediction. We expect that using multihead GATv2 and GraphNet architectures as GNNs for learning XAS spectra aligns with the essential understanding of the delocalized nature of molecular orbitals, which is crucial for accurate XAS prediction. Vaswani et al.^54^ have shown previously that multihead attention, incorporated in the multihead GATv2 model, can improve the performance of models by enabling them to attend to different parts of the input molecular graph simultaneously. Wiegreffe and Pinter^75^ have additionally shown that models that use the attention mechanism can provide better interpretability compared to nonattention frameworks, since they allow the visualization of which parts of the input are being attended by each head, making it easier to understand how the model is making predictions.

Thus, when it comes to XAS analysis, we can infer that the attention framework, which dynamically assigns importance weights to nodes surrounding a target node, yields superior attribution values compared to those of the static node-weighting scheme employed by the GCN framework. Moreover, combinatorial generalization in GraphNets, which enhances their ability to generalize and perform well on new, unseen graph structures and tasks, is crucial to their applicability to XAS predictions in diverse molecular structures. On the other hand, robustness and generalization in GraphNet models, which incorporate relational inductive biases, have achieved improvement compared to traditional GNNs such as GCNs, over a range of graph classification and regression tasks.^76−78^

### Robustness of the Explainability Performance of the GNN Models

**Figure 8:**
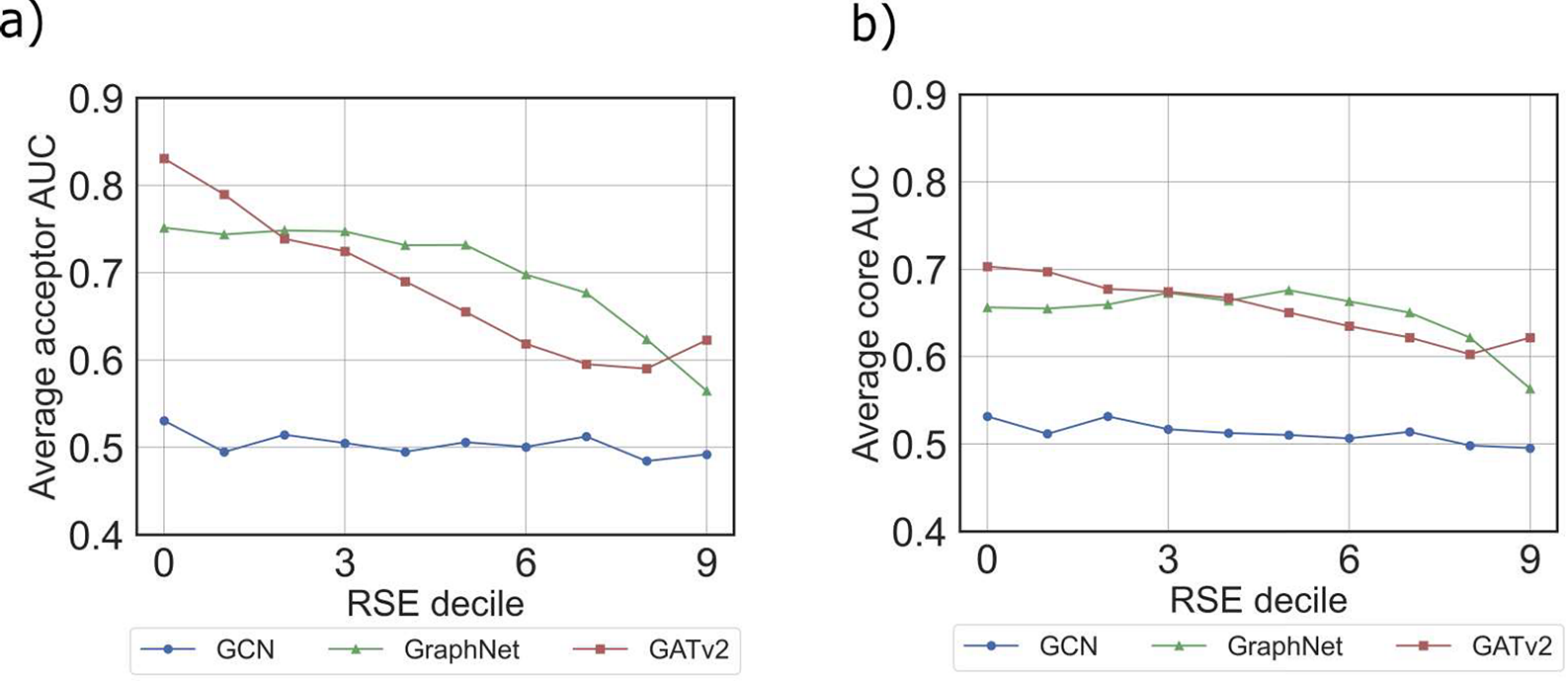
Variation of attribution AUC values for virtual (a) and core (b) orbitals with RSE decile values for three GNN models: GraphNet (green triangles), GATv2 (red squares), and GCN (blue points).

Having shown that CAM attributions allow the explanation of the individual peaks in predicted XAS, the next task is to determine how robust this explainability is with respect to the prediction accuracy itself and to the changes in the data set. To address the influence of prediction quality on interpretability, we first explored how the attribution AUC scores vary across different RSE values for the three GNN models. This is performed for each model by first distributing the molecules of the test data set into ten evenly large groups based on their RSE values. For these RSE deciles the average attribution AUC scores are computed and plotted in Figure 8 for both the virtual (a) and the core (b) orbitals. For the multihead GATv2 (red line) and the GraphNet (green line) models, the attribution AUC scores decline with increasing RSE values. The GATv2 sets on at the overall largest AUC of 0.83 (0.70) for the virtual (core) orbitals and then drops slightly below the GraphNet prediction to a value of about 0.6 (for both models). With the understanding that a larger counter of the RSE decile means a poorer prediction of the XAS spectrum, it becomes apparent that large AUC values are obtained when the overall spectrum prediction itself is reliable as well. Aligning this observation with the broader knowledge of quantum chemistry, we can infer that if ML predicts the spectrum more accurately, its understanding of orbital contributions improves correspondingly. In contrast, the GCN model’s average attribution AUC exhibits no variation across RSE deciles, staying close to the random baseline value of 0.5. This suggests that the model has a similar level of understanding of the ground truth for both strong and weak performances in XAS prediction, which was already explained by the ML quality of the GCN model in the last section.

**Figure 9:**
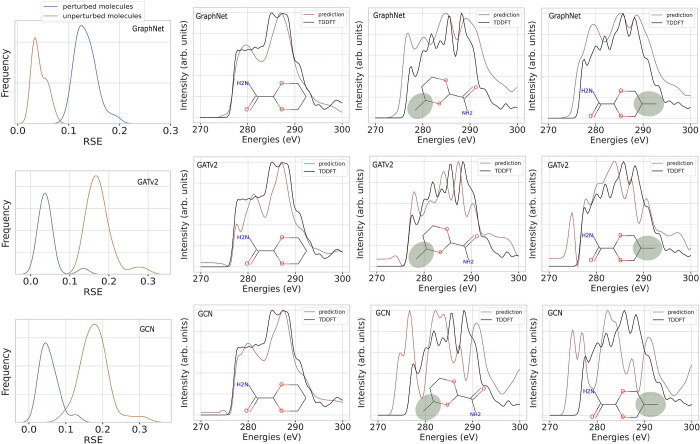
Impact of a perturbation through the replacement of functional groups. The left side of each row displays alterations in the RSE distribution for all GNN models when predicting the spectra for unperturbed structures selected from the data set (blue) and the perturbations of these structures (orange). Additionally, XAS spectra for different exemplary perturbations are shown (right), where a methyl group (highlighted in the gray circle) is added at different positions. The changed TDDFT spectra are shown in black, and their ML predictions are shown in red.

The robustness of model predictions (and their interpretations) usually decreases when there are biases in the training data set that the model erroneously learns.^38^ The QM9 data set is only a small representation of the vast chemical space of organic molecules and as such is biased toward molecules with certain functional groups. Furthermore, choosing a random subset of structures from this data set means that the resulting structures in the smaller QM9-XAS data set could also be further biased toward one or several types of functional groups. To identify whether such biases are learned by the model, one approach is to analyze the attributions of the model’s predictions and inspect whether CAM attributions are allocated to incorrect features of the input.^39^ In this case, the robustness of model predictions is tested by looking at how the model performance varies for predictions across similar chemical environments. The simplest way of doing so is by perturbing the chemical space around a molecule, e.g., by adding one or several functional groups at different places. We investigate the impact of the addition of one methyl group on randomly selected molecules from the test data set on both the attribution AUC and the RSE value obtained with the GNN models. For these novel 40 perturbed structures, XAS spectra were calculated as a reference for RSE determination using the same TDDFT method as above. Adding a methyl group at different positions in a molecule leads to changes in the TDDFT spectrum, as well as in the ML predictions as illustrated in the three right panels of Figure 9. The three GNN architectures respond differently to this change and give vastly varying predictions of the new spectrum, as indicated by their increased RSE values as well. Overall, the ML spectra deviate significantly from the TDDFT spectra. This difference in predictions across all molecules is summarized in the left panel of Figure 9, which illustrates the change in the RSE performance of the models for the 40 selected structures before and after perturbation. The RSE distributions of the unperturbed set of molecules have slightly different shapes for the different models, but all give mostly the same average RSE value of approximately 0.03. With the perturbation, the RSE of the GCN and the GATv2 both shift to an RSE average of 0.18, while the GraphNet model gives about 0.13. The altered RSE distributions of the perturbations of these structures clearly indicate a decrease in model performance for perturbed molecules, with the GraphNet model demonstrating a superior performance compared to the others. This difference indicates that the GraphNet model can generalize better to chemical environments that are rarely encountered in the data set and are less susceptible to biases.

The changes in the RSE are significant, even for the GraphNet model. This change can be attributed to the fact that when a methyl group is included and replaces a hydrogen atom, the size of the molecule increases. The largest molecules within the original QM9-XAS data set consist of a maximum of nine heavy atoms (C, N, O, F), while the perturbed structures, on average, contain more than nine heavy atoms. This increase in molecular size potentially represents outliers to the trained model, thereby leading to a decline in performance when predicting spectra.

**Figure 10:**
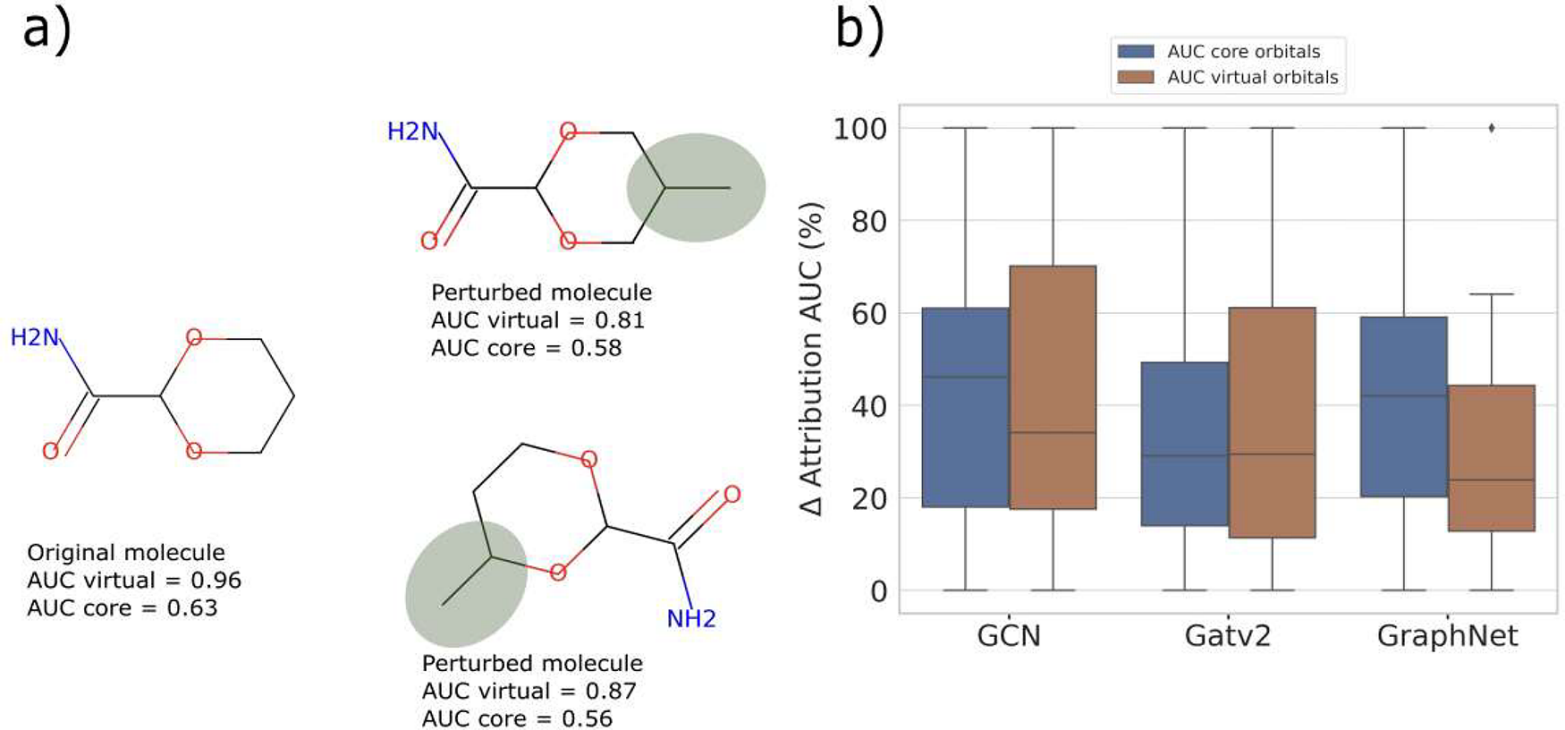
Attribution accuracy measured after perturbing random structures. (a) One specific molecular example to demonstrate the addition of −CH_3_ groups as perturbation along with the change of AUC values according to the GraphNet model. (b) Δ-AUC plots for the perturbed set of test molecules across the three GNNs.

Previous studies have demonstrated^35,38^ that when a model fails to learn the ground-truth logic, it can result in misplaced attributions and the misclassification of atoms within the molecule after perturbations. We therefore now look at how attribution AUC changes for the spectra of the perturbed structures when compared with the AUC values of the original molecules. Figure 10 shows the Δ-attribution AUC across all the models for the perturbed structures, where the Δ-attribution AUC is the percentage-difference in the attributions of the 40 perturbed structures compared to the AUC values of the unperturbed molecules. While the multihead GATv2 model shows a 30% decline in the attribution AUC of core orbitals after perturbation, GCN and GraphNet models experience over 40% change. In the case of virtual orbitals, GraphNet and multihead GATv2 models decrease by 25% and 30%, respectively, while the GCN model shows a 35% drop. The drop in relative attributions uniformly across all of the models aligns with the increase in RSE values for these molecules, discussed in Figure 9. Such large changes in both core and virtual orbitals in all GNN models can originate from the effects of changes in both local and global molecular features on the spectrum after perturbations which results in changes in atomic contributions to the peaks in the spectrum. Hence, while the local environment of an atom, which refers to the atoms in close proximity to the absorbing atom, strongly affects the spectral features in the XAS spectrum, the global environment of that atom and changes caused by perturbations can also play a significant role in determining the electronic structure and thus the final XAS spectrum. This is also in line with previous research which showed that the presence of long-range interactions between atoms, as well as the coordination number, chemical nature, and distance of these neighboring atoms, can have strong influences on the spectral features, such as the position and width of the XAS peaks.^8,79^ These findings demonstrate the importance of incorporating the local and global environment of nodes while learning structure–property relationships using GNNs.^80^

**Figure 11:**
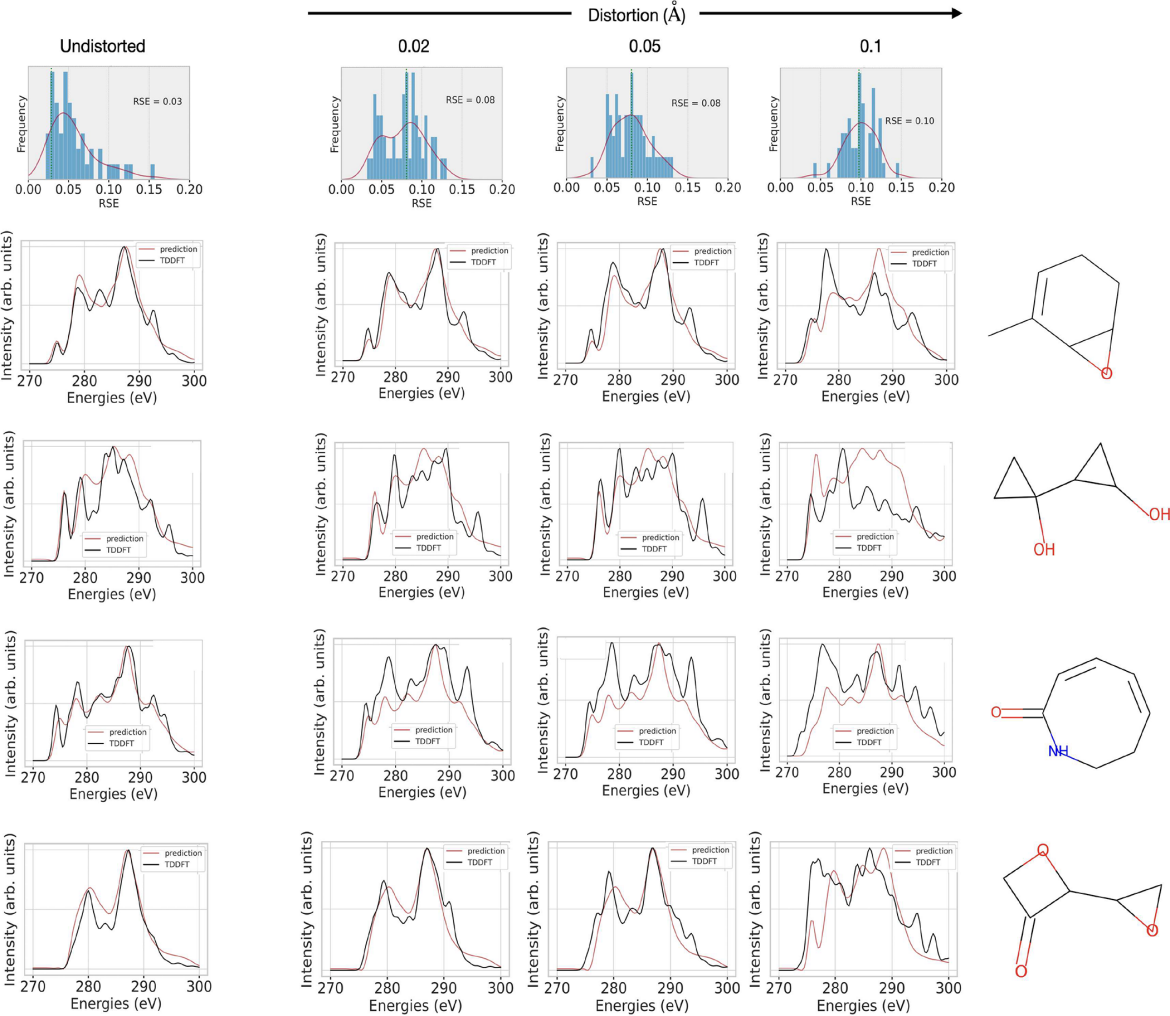
Evaluating the performance of the GATv2 model with structural distortions for four example molecules of the test set. The panels on the left give the TDDFT spectra (black) and the predicted spectra (red) for the undistorted case, while distortion is increasing for the three spectra on the right.

In addition, we examine whether the GNN model with best performance (i.e., GATv2) mimics the changes expected with structural distortions. To obtain distorted molecules, we choose a distortion parameter σ ∈ {0.02, 0.05, 0.1} Å to perturb randomly the atomic coordinates, i.e. in *x*-, *y*- and *z*-directions, in the respective molecule.^81^Figure 11 demonstrates how the TDDFT calculation and model prediction changes with respect to different distortion values. Already for the smallest distortion of only 0.02 Å, the TDDFT spectra change mostly in peak intensities and slightly in peak positions. These changes become more pronounced with stronger distortion. The model’s prediction of small distortions looks similar to the undistorted one, i.e., predicting the general features of the spectrum, which, however, results in an increasing RSE with increasing distortion. For the largest distortion of 0.1 Å, the model mimics more closely the changes of the TD-DFT spectra, while the RSE values increase again. Such changes in XAS spectrum prediction suggest that the representation of small molecular conformation changes would require more structural information in node and edge feature vectors beyond the bond lengths. This could be the atomic pairwise distances, dihedral angles, etc.

## Conclusion and Discussion

The aim of this work is to assist in the interpretation of peaks in X-ray absorption spectra (XAS) using a black-box machine learning (ML) method, i.e., graph neural networks (GNNs), as opposed to obtaining such information from purely conventional quantum chemical calculations. Yet, the underlying ground truth is based on the latter. In order to achieve this, we implement an explainability technique on various architectures of GNNs trained on a custom-developed carbon K-edge XAS data set of 65,000 small organic molecules, denoted as QM9-XAS, in which the molecules are a subset of the original QM9 data set.

The main difficulty in explaining properties with GNN models, as complex as the physical origin of peaks in XAS spectra already is, is the inherent lack of knowledge about the internal mechanisms of the model and how to correlate the properties of the model with the knowledge gained from quantum chemical calculations. We devised an approach that reflects a chemist’s understanding of the XAS phenomenon as electronic excitations originating from individual atoms, which treats the underlying excitations of XAS peaks as a linear combination of core-to-valence orbital transitions and calculates the contribution of an individual atom to the participating core and valence orbitals. This produces atom labels denoting whether a particular atom contributes to an XAS peak within a specified energy range, allowing for the acquisition of the chemical ground truth and assessment of the extent to which an ML model comprehends the XAS spectra.

The rationale behind peaks observed in ML-predicted XAS spectra is unraveled via the so-called class activation map (CAM) attributions, highlighting the importance of individual nodes (atoms) in a molecular graph to the target peak of the spectrum. For a quantitative assessment of the graph attributions, we characterize the true and false positive rates of CAM attributions by calculating the area under the curve of the receiver operating characteristic (AUC-ROC), which is effectively a measure of how well the node attributions match the atomic contributions from the ground truth. Through this comparison between the chemical ground truth, i.e., here the core-to-valence orbital transitions, and CAM attributions, we demonstrate that while it is important to consider the overall performance of the GNN model in accurately predicting XAS features, the degree of explainability of the different architectures of GNN models differentiates them. We find that GNN models such as GraphNet and multihead GAT layers, which are in principle able to capture both the local and the more global chemical environment of an atom in a molecule, not only perform well in their spectra predictions but also the explanations obtained from these models are consistent with the quantum chemical interpretation of XAS.

To examine model robustness, we add a methyl group as a perturbation to a random set of molecules of the test data set of QM9-XAS. A decrease in performance is observed for all GNN models, with the GraphNet model showing the least decrease in performance, as assessed by the increase in relative spectral error (RSE). We suspect that the differences in the learning mechanisms between the three GNN architectures used have a significant effect on the changes in the RSE distribution and AUC attributions. The observed changes in attribution AUC highlight the limitations of relying only on the prediction accuracy obtained on a test data set to evaluate the performance of a model.

In conclusion, the approach presented here provides a recipe for incorporating explainability into GNN models using custom-generated data, which provides insight into the physical origin of spectroscopic predictions. Although the GNN models in this work are trained to predict the entire XAS spectrum, the model’s attributions provide an opportunity to obtain some insights into local XAS spectra, i.e., for individual carbon atoms, with cost-effective computational resources. While our framework was demonstrated for carbon K-edge XAS prediction, the approach can be easily extended to other energy regimes, such as nitrogen and oxygen edges of molecules and metal complexes or even other spectroscopic techniques. Further, since this approach relies on theoretical data obtained from quantum chemical calculations, it can also be used to obtain ground-truth data for models trained on experimental data.

Direct comparison of predictions made in this approach to experimental spectra is challenging due to several factors influencing the experimental observations including solvent effects, experimental conditions like temperature and pressure, and structure-determining factors such as coexistence of multiple metastable conformers contributing to the experimental spectra. Incorporating these effects is often not so trivial using the existing theoretical approaches, and thus, corrections to theoretical spectra are necessary, often done on a case-by-case basis, depending on the molecular system and its environment. Considering the configurational phase space of the molecule in data set generation for training the model is one of the ways one can improve the discrepancy between a model’s prediction and experimental spectra. For large molecular structures such as proteins and nanoparticles, computation of spectra at *ab initio* level of theory is often a challenge, although their XAS spectra can give insights into their different local environments.

While traditionally these have been tackled by the use of fingerprints determined on an ad-hoc basis, we believe that the development of more sophisticated and efficient machine learning frameworks, while maintaining explainability, offers a promising avenue for predicting spectra at low costs as well as getting insights into local molecular environments.

## Data Availability

The code used to train the models and generate the figures in this publication is publicly available at https://github.com/AI-4-XAS/XASNet-XAI. The QM9-XAS data set is available at 10.5281/zenodo.8276902.
